# Structure, function, and plasticity of hippocampal dentate gyrus microcircuits

**DOI:** 10.3389/fncir.2014.00107

**Published:** 2014-09-10

**Authors:** Peter Jonas, John Lisman

**Affiliations:** ^1^IST Austria (Institute of Science and Technology Austria)Klosterneuburg, Austria; ^2^Biology, Volen National Center for Complex Systems, Brandeis UniversityWaltham, MA, USA

**Keywords:** hippocampus, dentate gyrus, granule cells, mossy cells, mossy fibers, mossy fiber synapses, adult neurogenesis

The hippocampus mediates several higher brain functions, such as learning, memory, and spatial coding. The input region of the hippocampus, the dentate gyrus, plays a critical role in these processes. Several lines of evidence suggest that the dentate gyrus acts as a preprocessor of incoming information, preparing it for subsequent processing in CA3. For example, the dentate gyrus converts input from the entorhinal cortex, where cells have multiple spatial fields, into the spatially more specific place cell activity characteristic of the CA3 region. Furthermore, the dentate gyrus is involved in pattern separation, transforming relatively similar input patterns into substantially different output patterns. Finally, the dentate gyrus produces a very sparse coding scheme in which only a very small fraction of neurons are active at any one time.

How are these unique functions implemented at the level of cells and synapses? Dentate gyrus granule cells receive excitatory neuron input from the entorhinal cortex and send excitatory output to the hippocampal CA3 region via the mossy fibers. Furthermore, several types of GABAergic interneurons are present in this region, providing inhibitory control over granule cell activity via feedback and feedforward inhibition. Additionally, hilar mossy cells mediate an excitatory loop, receiving powerful input from a small number of granule cells and providing highly distributed excitatory output to a large number of granule cells. Finally, the dentate gyrus is one of the few brain regions exhibiting adult neurogenesis. Thus, new neurons are generated and functionally integrated throughout life. How these specific cellular and synaptic properties contribute to higher brain functions remains unclear.

One way to understand these properties of the dentate gyrus is to try to integrate experimental data into models, following the famous Hopfield quote: “Build it, and you understand it.” However, when trying this, one faces two major challenges. First, hard quantitative data about cellular properties, structural connectivity, and functional properties of synapses are lacking. Second, the number of individual neurons and synapses to be represented in the model is huge. For example, the dentate gyrus contains ~1 million granule cells in rodents, and ~10 million in humans. Thus, full scale models will be complex and computationally demanding.

In this Frontiers Research Topic series of papers, we collect important information about cells, synapses, and microcircuit elements of the dentate gyrus. We have put together a combination of original research articles, review articles, and a Methods article. The collection includes contributions on:

- Connectomics and lamellar organization of the dentate gyrus (Sloviter and Lømo, 2012).
- Role of hilar mossy cells in dentate gyrus microcircuits (Jinde et al., 2013; Scharfman and Myers, 2013).
- Structural and functional properties of granule cell axons and mossy fiber output synapses (Zhao et al., 2012; Ruiz and Kullmann, 2013).
- Adult neurogenesis, with focus on integration of new granule cells into the dentate network (Lopez et al., 2012; Dieni et al., 2013; Vivar and van Praag, 2013).
- Analysis of coding mechanisms by full-scale computational models and theoretical approaches (Schneider et al., 2012; Stella et al., 2013).
- Plasticity of the dentate gyrus during injury (Perederiy and Westbrook, 2013).

We hope that the collected information will be useful for both experimentalists and modelers. We also hope that the papers will be interesting beyond the small world of “dentology,” i.e., for scientists working on other brain areas. Ideally, the dentate gyrus may serve as a blueprint, helping neuroscientists to define strategies to analyze network organization of other brain regions.

## Conflict of interest statement

The authors declare that the research was conducted in the absence of any commercial or financial relationships that could be construed as a potential conflict of interest.
